# The Evolution of the Ribosome and the Genetic Code

**DOI:** 10.3390/life4020227

**Published:** 2014-05-20

**Authors:** Hyman Hartman, Temple F. Smith

**Affiliations:** 1 Earth, Atmosphere, and Planetary Science Department, Massachusetts Institute of Technology, Cambridge, MA 02139, USA; 2 BioMedical Engineering, Boston University, Boston, MA 02215, USA; E-Mail: templesmith1@comcast.net

**Keywords:** Genetic Code, evolutions, ribosomal proteins

## Abstract

The evolution of the genetic code is mapped out starting with the aminoacyl tRNA-synthetases and their interaction with the operational code in the tRNA acceptor arm. Combining this operational code with a metric based on the biosynthesis of amino acids from the Citric acid, we come to the conclusion that the earliest genetic code was a Guanine Cytosine (GC) code. This has implications for the likely earliest positively charged amino acids. The progression from this pure GC code to the extant one is traced out in the evolution of the Large Ribosomal Subunit, LSU, and its proteins; in particular those associated with the Peptidyl Transfer Center (PTC) and the nascent peptide exit tunnel. This progression has implications for the earliest encoded peptides and their evolutionary progression into full complex proteins.

## 1. Introduction

The Genetic Code is at the heart of molecular biology. One great problem facing the molecular biologist is the Origin and Evolution of the Genetic Code. It is important to separate this overall problem into two sub-problems: The Origin of the Genetic Code and the Evolution of the Genetic Code. This allows one to focus on the problem of the evolution of the Code from its earliest form to its extant form. This can be done by studying the structures and sequences of the ancient universal proteins and RNAs found in the translational complex. 

**Figure 1:**
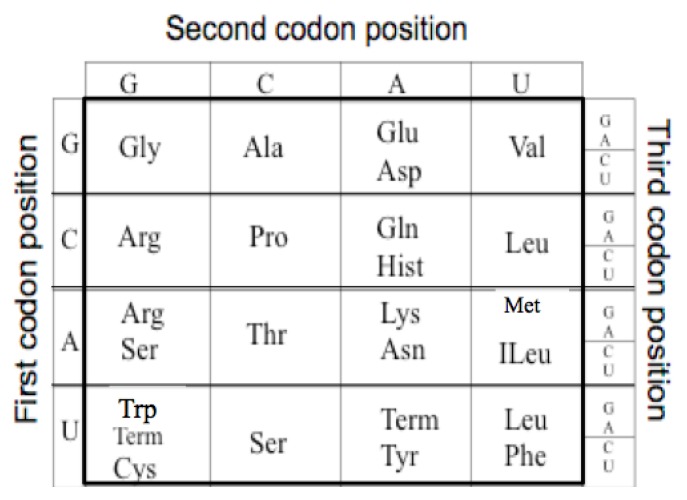
The Genetic Code. The table has been reoriented Displaying the four RNA nucleotides in the non-standard order, Guanosine monophosphate, G; Cytosine monophosphate, C; Adenosine monophosphate, A; and Uridine monophosphate, U; to encode the 20 amino acids: Glycine (Gly), Alanine (Ala), Arginine (Arg), Proline (Pro), Threonine (Thr), Serine (Ser), Cysteine (Cys), Glutamic Acid (Glu), Glutamine (Gln), Aspartic Acid (Asp), Asparagine (Asn), Lysine (Lys), Histidine (Hist), Valine (Val), Leucine (Leu), Isoleucine (ILeu), Methionine (Met), Phenylalanine (Phe), Tyrosine (Tyr), Tryptophan (Trp) and the peptide termination codons, Term.

The extant Genetic Code is a mapping from an alphabet of four nucleotides (GCAU) to the 20 amino acids. As there are four nucleotides and twenty amino acids, the minimum mapping is, thus, between the 64 triplet nucleotide codons (4 × 4 × 4) and the twenty amino acids. For example GGG codes for Glycine see Figure 1.

The code has a particular degenerate structure, which resides in the third codon nucleotide position. Some of the amino acids, such as Glycine, Alanine, Arginine, Proline, *etc*., have four synonymous codons allowing any of the four nucleotides in the third position. Other amino acids, such as Glutamic acid, Aspartic acid, *etc.*, have a codon differing only in the third position with either a purine or a pyrimidine nucleotide. In addition the second positions in the Code Table are correlated with some amino acid biochemical properties, such as hydrophobicity seen in the U column. 

## 2. Amino Acids Metabolic Metric

Granick [1] proposed that *Biosynthesis recapitulates Biopoesis.* Combining this with the universal and proposed early nature of the citric acid cycle [2,3], suggested a metabolic metric. Using the number of catalytic steps in the biosynthesis of each amino acid from acetate, glyoxalate, pyruvate, and the rest of the citric acid cycle**.** This metric can be used as one estimates as to when an amino acid entered the Genetic Code. For example, Alanine, Glycine, Aspartic acid, Glutamic acid would be early candidates by this metric as they are only one catalytic step away from the Citric Acid cycle. Many of these catalytic steps from the Citric Acid Cycle are transaminations, which are mediated by the coenzyme Pyridoxal Phosphate, PLP, as in the transamination of Pyruvate to Alanine, Oxalacetate to Aspartate, Alpha-ketoglutarate to Glutamate, and Glyoxalate to Glycine. This implies that the coenzyme PLP may be ancient and have preceded the associated enzyme [2].

The next group of amino acids by this metric would be Glutamine, as it is one step from Glutamic acid, Asparagine, as it is one step from Aspartic acid, and Serine, as it is one step from Glycine. Then there would be Proline, as it is two steps away from Glutamic acid. The amino acid cysteine is two steps from Serine. Next would be Threonine, which is three steps from Aspartic acid. The other amino acids, such as Valine, Leucine, Isoleucine, Methionine, Phenylalanine, Tyrosine, and Tryptophan, require many more catalytic steps from the Citric Acid cycle. Lysine and Arginine by the metabolic metric would also be expected to be late additions to the Genetic Code. Histidine is unique as its biosynthetic pathway is close to that of the nucleotide bases and thus a metabolic measure from the Citric Acid Cycle is not directly comparable with that of the other amino acids.

The coevolution theory [4] considers that there are two sets of amino acids, 10 prebiotic amino acids and 10 metabolically derived amino acids. This is a mixed theory as 10 amino acids are found in a prebiotic soup [5,6]. The only overlap between these two metabolic theories is found in the pairs Glu-Gln and Asp-Asn.

## 3. The tRNA and Aminoacyl-tRNA Synthetases

**Figure 2:**
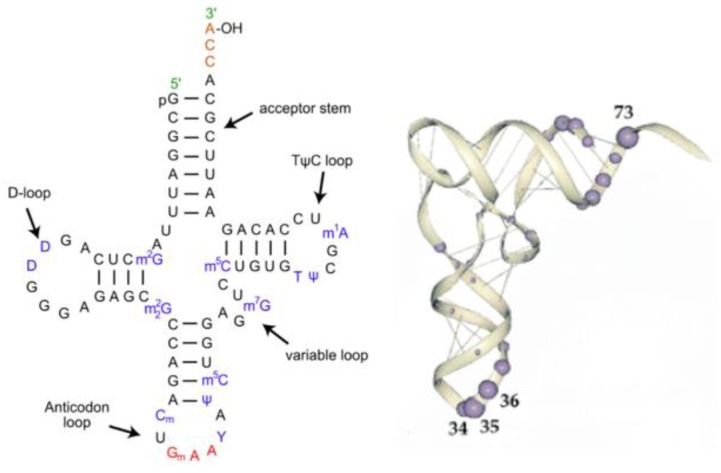
The tRNA structure. The Left-hand side is a representation of tRNA’s secondary structure. The stem loop and acceptor arm contain invariant GC nucleotides, as does The D loop, and the TΨCG loop and the UCCA of the acceptor arm. The Right-hand side is an RNA backbone representation of the 3D folded structure, with the most common Aminoacyl-tRNA synthetase contact base positions highlighted.

The first step in the implementation of the extant Genetic Code is the attachment of an amino acid to a transfer RNA (tRNA) with the correct anticodon. This then allows the association of each amino acid to its correct codon in the messenger RNA (mRNA). The structures of most tRNAs have the cloverleaf secondary structure shown in Figure 2. There are three helical stem loop structures labeled by their loops: the anticodon stem loop; TΨC stem loop and the D stem loop. The upper helical stem is the acceptor arm ending with the amino acid acceptor four nucleotides 3′XCCA. These secondary structures fold into an L-shape shown on the right side of Figure 2. The most relevant bases recognized by the Aminoacyl-tRNA synthetases are the three nucleotides in the anticodon loop and the six bases in the double helix in the acceptor arm and the base in position 73.

In the folded tRNAs (Figure 2), the amino acid acceptor arm is at one end of the “L” and the anticodon loop is at the other end. The D and the TΨCG loop fold form the corner of the “L”. The separation between the acceptor site and the anticodon loop is reflected in the interaction between the tRNA and the Aminoacyl-tRNA synthetase structures. Most synthetases have two recognition sites, one for the anticodon loop and the other binding to the acceptor arm plus the 5′*CCA3′ end. The latter end of the synthetase is the catalytic domain of the synthetase that attaches the amino acid to the ribose of the Adenosine at the *CCA end of the acceptor arm of the tRNA.

There are twenty Aminoacyl-tRNA synthetases, one for each of the twenty canonical amino acids, with particular rare exceptions [7]. They form two Classes defined by their catalytic domains. One Class has a Rossman fold catalytic domain and is designated Class I. The other Class is designated Class II and has a fold found in Biotin Carboxylase. Both catalytic domains carry out the following two reactions in attaching each amino acid to the correct tRNA:

amino acid + ATP → aminoacyl-AMP + PP_i_

aminoacyl-AMP + tRNA → aminoacyl-tRNA + AMP

**Figure 3:**
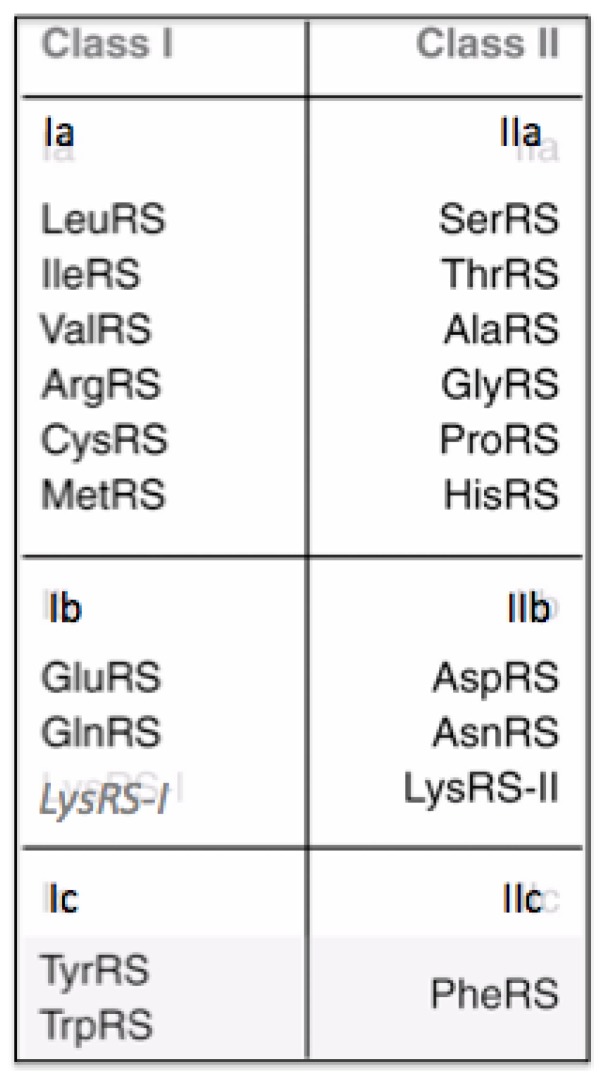
The 20 amino acids and their associated Aminoacyl tRNA synthetases. Lysine appears twice however the Class I version only appears in a few organisum in which there is not a Class II version.

There are ten amino acids in each of the two Classes, shown in Figure 3. Lysine is unique among the amino acids as it occurs in Class I and Class II, however, not in the same organism. When it occurs in an organism associated with a Class I Aminoacyl-tRNA synthetase, there are eleven amino acids in I and nine in Class II. There are modifications to the number of Aminoacyl-tRNA synthetases in a number of organisms. However, it is the general case that there are 20 Aminoacyl-tRNA synthetases.

It has been concluded “that Class II Aminoacyl t-RNA synthetases (are) mostly associated with the primordial amino acids, while Class I are more related to amino acids that were added later” [8]. The idea that the Class II synthetases are older implies that an earlier Genetic Code could have encoded Gly, Ala, Pro, Thr, Ser, Asp, Asn, Lys, His, and Phe. However from the metabolic metric’s logic, Histidine, Phenylalanine, and Lysine would have been later amino acids. One could also assume that since Aspartic acid is a biochemical precursor of Asparagine it is likely to have preceded it in the Genetic Code. One is left with Gly, Ala, Pro, Thr, Ser, and Asp as early probable encoded amino acids from both the metabolic metric and the assumed older nature of the Class II Aminoacyl-tRNA synthetases. 

In 1988, Hou and Schimmel [9] reported that a GU base pair in the acceptor arm of the tRNA was sufficient for the Alanyl-tRNA synthetase to add alanine to its cognate tRNA, and later, related this to its position relative with the neighboring GC pair [10]. In 1989, Franklyn and Schimmel reported that Alanine was specifically joined by its Alanyl-tRNA synthetase to an RNA microhelix containing only its normal tRNA adaptor stem but not its anticodon. This ability was extended to more amino acids: five amino acids in Class II (Gly, Ala, Ser, Asp, and His) and three amino acids in Class I (Val, Ileu, and Met) [11]. This meant that in the acceptor arm there was a nucleotide code that could be recognized independently of the anticodon. This code was called the Operational Code. 

This Operational code was shown to be located in the first three base pairs of the acceptor arm and the unpaired nucleotide at position 73 of the t-RNA. In 1997, Rodin and Ohno pointed out in their paper that “In contrast with anticodons, which are built of the four nucleotide bases, G, A, C, and U, their double-stranded precursors, the operational code, in the 1-2-3 positions of the tRNA acceptor arms appear as triplets almost invariably composed only of G-C and C-G base pairs” [12]. This led them to postulate an early GC operational code in the first three base pairs of the acceptor arm. They considered this primitive GC code to encode Glycine, Alanine, Proline, and Arginine. Note that Glycine, Alanine, and Proline are suggested above as among the likely early entrants into the Genetic Code. However, Arginine is not considered to be among the early encoded amino acids as it is eight catalytic steps from Glutamate and is associated with the Class I Aminoacyl-tRNA synthetases. However, assuming that early encoded peptides needed to interact with RNA, there is a question as to whether Arginine or even Lysine were preceded by an earlier and metabolic simpler positively charged amino acid. The issue with Arginine and its association with a potential early CG code is discussed in a following section on the large ribosomal proteins.

Paul Schimmel and co-workers [13] and especially Rodin and Ohno [12] established that there was an operational GC code in the acceptor stem of the tRNA. However, it is not only in the acceptor arm where the ancient GC operational code is to be found, but also in the loops of the extant tRNA.“If one considers the cloverleaf structure of t-RNA, then aside from the anti-codon loop, the major invariances of the stem loops are 5′UGGU3′, 5′UCGA3′, 5′*CCA3′. The GG, CG, CC are considered to be remnants of the ancient doublet GC (operational) code. Furthermore the modern tRNA is a “tetramer (arm and loop structure) and it is in the loops that the evolution of t-RNA can be followed” [13]. Based on the charging of the micro helices the minimal size of the proto-tRNA was a likely 17-nucleotide stem loop microhelix (seven nucleotides in the loop and ten in the double helical arm and in the amino acid attachment region four nucleotides (3*′*XCCA). Furthermore the cloverleaf tRNA can be considered a tetramer ligated from at least three microhelices. 

It is clear that there was a synergetic coevolution of the genetic code and tRNA synthetases. Thus, we are assuming the existence of some form of a very early tRNA amino acid charging system, a proto synthetase. It has been proposed that the tRNA synthetase evolution began with peptides formed in a “Thioester World” preceding those encoded by the earliest RNA based system. The evidence for this evolution is found in the catalytic site of the Class II synthetases [14] and the thioesters formed at the editing modules of the Class I synthetases [15]. “These and other data support a hypothesis that the present day aminoacyl-tRNA synthetases originated from ancestral forms that were involved in noncoded thioester-dependent peptide synthesis, functionally similar to the present day non-ribosomal peptide synthetases” [15]. The source of amino acids in a Thioester World may have been synthesized unrelated to citric acid cycle, e.g., abiotically and, thus, not subject to the Metabolic matrix. Such amino acids could have formed early non-coded peptides as well. These issues need to be dealt with in relation to the Origin of the Genetic Code rather than here with its evolution.

## 4. The Universal Ribosomal Proteins

The ribosome comes in two subunits, the Large Subunit, LSU, and the Small Subunit, SSU. Each subunit is mainly a complex of one major RNA and a set of ribosomal proteins. For example, in Bacteria (*E. coli*) the SSU rRNA is 1542 nucleotides long and the LSU’s major rRNA is nearly 2906 nucleotides long, with an additional 5S RNA in the LSU. 

Carl Woese and co-workers [16] exploited the deep evolutionary information found within the ribosomal RNA. From which, they were able for the first time to discern that there were three cellular domains of Life namely the Bacteria, the Archaea, and the Eukarya. This discovery was made by an intensive phylogenetic study of the sequences and secondary structures of the SSU rRNA from many organisms [16] and [17]. While much of the rRNA structure is common to all three phylogenetic domains, there are subdomains of the rRNA, *i.e*., segmental structures unique to each. This is not dissimilar to what is seen in the universal ribosomal proteins, discussed below.

There are 78 Eukaryotic Ribosomal Proteins, 68 in Archaeal ribosomes and 57 in Bacterial ribosomes. When these ribosomal proteins are aligned across the Archaea and Eukarya, all of the 68 Archaeal ribosomal proteins are homologs to 68 of the 78 eukaryotic ribosomal proteins [18]. There are an additional ten unique Eukaryotic ribosomal proteins belonging to the Eukaryotic signature proteins [19]. The Eukaryotic ribosome, from the point of view of these ribosomal proteins, is an Archaeal ribosome with a small number of additional ribosomal proteins. When the 68 Archaeal ribosomal proteins are aligned with the 57 Bacterial ribosomal proteins, only 34 are found to be homologous to those of Archaea and the Eukarya. These 34 proteins are, thus, defined as Universal Ribosomal Proteins, 15 in the SSU and 19 in the LSU. 

## 5. The 15 SSU Ribosome Proteins

**Figure 4:**
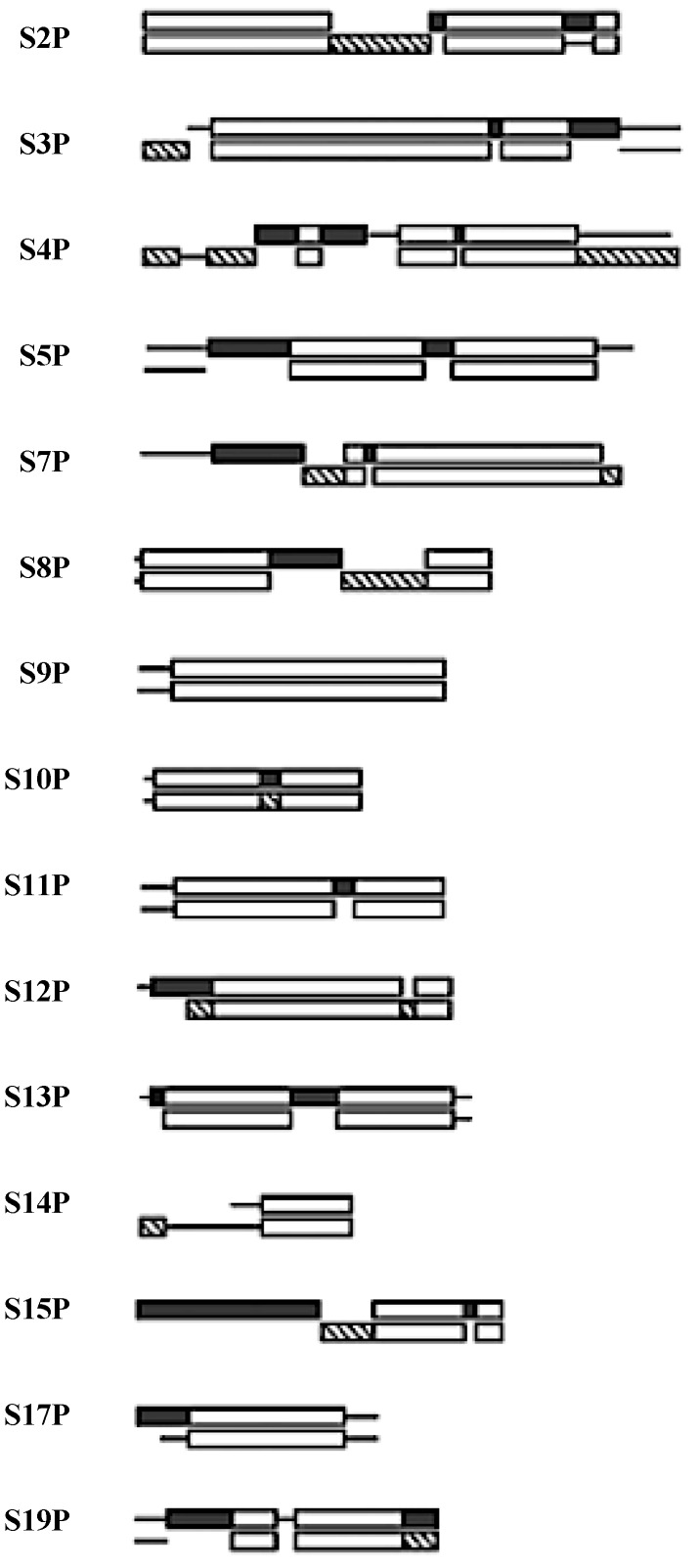
The SSU protein block structure. Universal peptides blocks are in white, the Bacterial blocks (**bottom**) are in hashed and the Archael (**top**) blocks in black [21].

A quick review of the 15 SSU universal ribosomal proteins shows many similarities to those of the LSU. They have functions involved in: sequential folding of the SSU rRNA; stabilization of the folded SSU; and the initiation of the binding of the SSU to the large subunit of the ribosome [20]. They have a taxonomic sequence block structure [21] and a mix of globular and globular plus extensions domain structures. See Figure 4.

**Figure 5:**
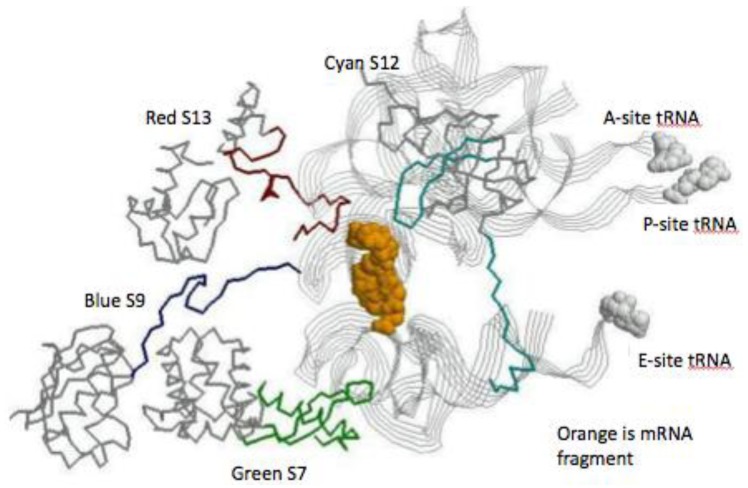
The SSU decoding site. The view is from just below the mRNA’s (space fill orange) looking up toward the tRNA contacts. The three tRNAs are displayed as gray ribbons, with space fill attached amino acids. The globular protein domain backbones are in gray and their extension are: S7 green, S9 blue, S12 cyan, and S13 red.

Five of the 15 universal SSU proteins (S2, S3, S4, S14, S15) are globular in structure while the other ten (S5, S7, S8, S9, S10, S11, S12, S13, S17, S19) have peptide extensions in addition to their globular portion. The majority of the globular domains are found on the SSU surface while the extensions reach into the ribosome’s interior [22]. Six of these universal proteins (S5, S7, S8, S10, S12, S17) have hairpin extensions while S9, S11, S13, S19 have N- and/or C-terminals extensions. The extensions of S7, S9, S12, and S13 are found at the decoding site of the SSU, see Figure 5. 

Since the taxonomic block structure has suggested that these proteins may have been originally assembled from peptide segments [23] one is curious as to any relationship between the taxonomic block sequence structure and the above active site extensions. While the lengths of these extensions, particularly the terminal ones, vary somewhat within a given taxonomic kingdom, all of the SSU active site extensions overlap sequence blocks common to all three taxonomic kingdoms. 

## 6. The 19 Universal LSU Proteins

Many of the LSU 19 universal ribosomal proteins share a globular plus nonglobular extension domain structure. Extensions here refer to segments of these proteins that extend away from the more compact globular domain for a significant distance. The vast majority of these LSU protein globular domains are found on the subunit’s surface. For example, L5, L11, L18, L24, L29p, and L30 are nearly pure LSU surface proteins [24]. The L10e/L16 LSU protein is an exception in having its globular domain largely buried in the interior of the LSU. L10e/L16 has an elongated globular structure that approximates the size of an RNA helix. Since it lies parallel to the two PTC active site large RNA helices, it seems to mimic a third RNA helix buried within subunit [23]. L2, L15, L18, and L24 have in addition to their globular, a long N-terminal non-structured extension. Proteins L2, L3, L4, L5, L13, L14, and L22 have extensions that are primarily hairpin or loops extending from within their globular domains. Like many ribosomal proteins L3’s and L4’s extensions reached deep into the rRNA structure, providing extensive RNA contacts. 

The antiquity of the above 19 ribosomal proteins is not only supported by their homology across all three kingdoms, but by their taxonomic block structure. That is, there are segments or alignment blocks common to all three kingdoms, while there are others common to only Bacteria or Archaea/Eukarya [21,25], This feature has been used to suggest that these proteins date to a very early time when proteins were assembled from relatively short peptides. These block structures also suggest that the globular portion *versus* the peptide extensions of these universal ribosomal proteins are also related to how the ribosomal proteins were assembled from their peptides.

## 7. The LSU Universal Ribosomal Proteins

A number of recent papers on the Origin and Evolution of the Ribosome concluded that the LSU of the ribosome was older than the SSU [23,26,27,28]. This makes sense if one assumes that the Peptidyl-transferase activity allowing for the early simply coded formation of short peptides (the Operational Code) came before the complexity of SSU and mRNA decoding. This in turn implies that at least some of the LSU protein peptides are older than those of the SSU and may still contain early peptide coding information. This conclusion was supported by a recent paper entitled *Inferring the Ancient History of the Translation Machinery and Genetic Code via Recapitulation of Ribosomal Subunit Assembly Orders*—“The overall trends of amino acid usage across the assembly maps of the LSU and SSU are most congruent with an evolutionary history in which the initial protein component of the LSU predated that of the SSU” [29].

In comparison to the LSU with its PTC-associated rRNA [23], the decoding site of the SSU has no discernable self-folding RNA center about which the SSU might have evolved by simple expansion. The evolution of the SSU about the decoding site is more complicated. In fact, it is even possible that it was involved in the evolution of some kind of RNA replicase [30]. The SSU decoding site contains associated peptide extensions that primarily make contact with the tRNAs and the messenger RNA rather than with the rRNA. Thus, without a good model for the evolutionary sequence of the SSU RNA, we concentrated on the evolution of the LSU and its Universal ribosomal proteins.

The early ribosome is believed to have evolved about the LSU active site, the Peptidyl Transfer Center (PTC) core [27,28,31]. The four LSU universal proteins, L2, L3, L4, and L22 have extensions reaching into this PTC and are involved in the early large subunit folding as well. Fox [32], thus, proposed that L2, L3, L4, and L22 are likely to contain the earliest peptides of the translational apparatus. Even more recently, in a major effort to model the ancestral ribosome, Hsiao *et al.* [33] demonstrated that the extensions of L2, L3, L4, L15, and L22 would all react with their LSU rRNA core model and they co-precipitated with said core. 

**Figure 6:**
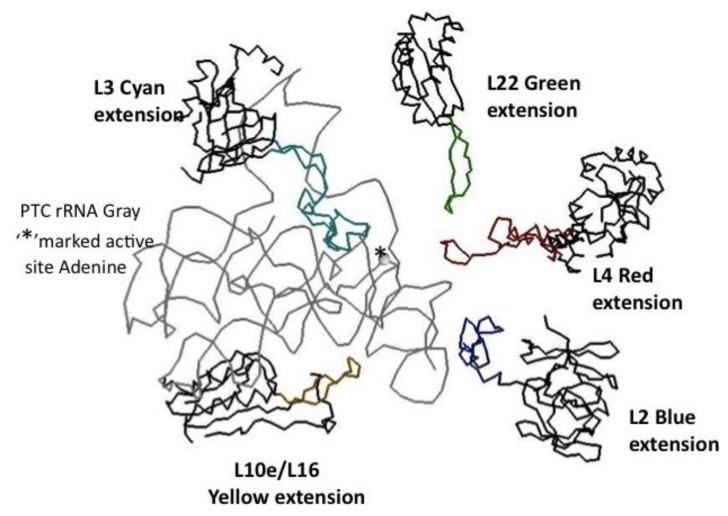
The minimum PTC and associated proteins. The PTC rRNA backbone is displayed in gray, the catalytic Adenine of the active site is marked with an asterisk. The five closely associated ribosomal proteins’ globular domain backbones are in black and their extensions in color: L2 blue, L3 cyan, L4 red, L10e/L19 yellow, and L22 green. The coordinates are from the X-ray Archaeal LSU structure, 1JJ2.pdb.

**Figure 7:**
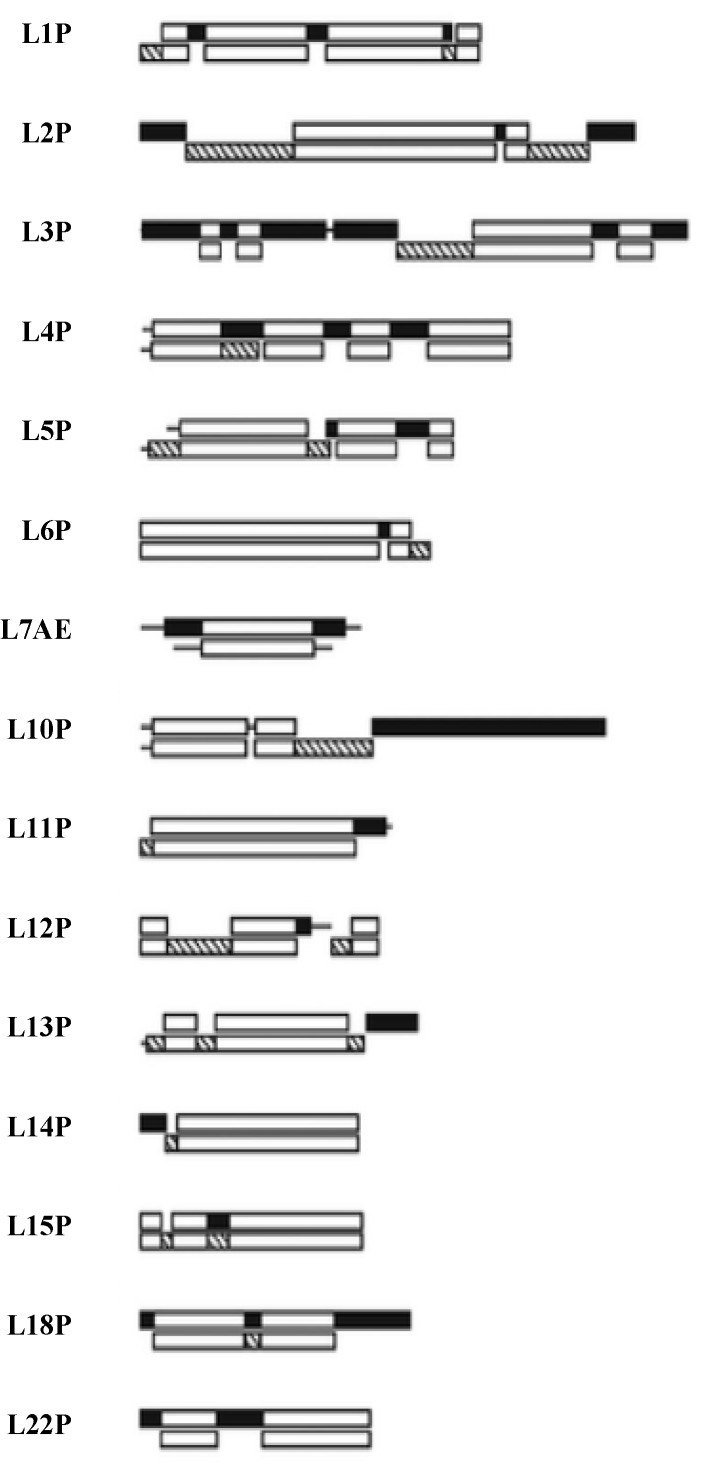
The LSU Protien block structure. Universal peptides blocks are white, the Bacterial blocks are hashed and the Archael ones in black [21].

The LSU protein extensions L4 and L22 form loops or hairpin structures. L4 (42–100) forms a loop with a series of beta turns. L22 (77–102) forms a hairpin structure with a near Classic beta turn. Both L4 and L22’s extensions reach in toward the PTC active site from the direction of the newly synthesized peptide exit tunnel (Figure 6). L3 (204–261) has a complex loop with double “headed” extreme, each having a beta turn-like end. L3’s large extension makes extensive contact with the rRNA helices forming the “floor” of the PTC. L3 also has a terminal more linear extension interacting with an RNA loop protruding above one of these RNA helices. L2 (196–237) on the other hand, has a C-terminal extension that approaches the PTC from the opposite side from that of L3’s extension. Interestingly this L2 extension approaches the PTC from above and gets closest to the extension of L4 (Figure 6). All of these LSU extensions have been shown to be contained in universal blocks as per Figure 7 [21].

The extensions of L3 and L22 are totally contained within a sequence segment or alignment blocks recognized as universal among all three kingdoms, Bacteria, Archaea, and Eukarya. L2’s extension sequence is also universal except for the very C-terminus, which differs between Bacteria and Archaea. L4 initially appears more complex, while most of this extension is clearly contained within a universal sequence block, the mid 20 amino acids do not align between Bacteria and Archaea. However, even these two mid-segments have nearly an identical three-dimensional structure and rRNA contacts, as well as having in their terminal contacting beta turns identically placed Glycine residues. Thus, in this case one can assume these extensions are universal, representing very ancient universal peptides with structure and rRNA contacts conserved if not full sequences.

The taxonomic sequence block structure of the Universal LSU ribosomal proteins and their extension structure supports the idea that these earliest encoded proteins were likely only relatively short peptides and that their more organized globular domains came later. It could be concluded that the extensions of these LSU proteins are associated with the PTC (LSU core) and are involved in LSU early folding, that they likely represent the very oldest coded peptides selected for their ability to stabilize the RNA structure involved in their own synthesis. Their universal nature, their association with the PTC and its assumed antiquity strongly support that they are representatives of, as Fox [32] has suggested, the very oldest peptides. That there is “fossil” information in the ribosomes has been noted earlier [34], however, with a focus on the total rRNA.

**Figure 8:**
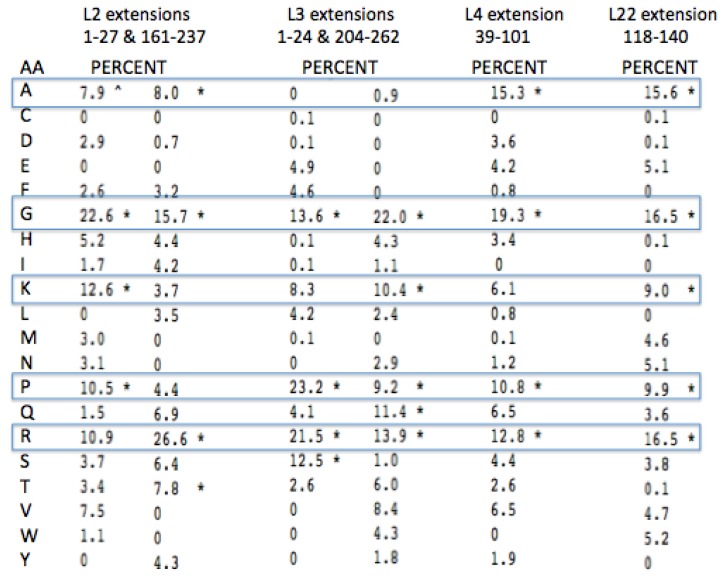
Peptidyl Transfer Center (PTC)-associated protein extension amino acid conservation. The pairwise amino acid conservation as a percentage of all common alignment pairs conserved among 200 aligned pairs of distantly related Archaea LSU proteins. The extensions were defined from the coordinates from the X-ray Archaeal LSU structure 1JJ2.pdb. The asterisk, * marks the top four highest percentages for each protein among the aligned extensions.

**Figure 9:**
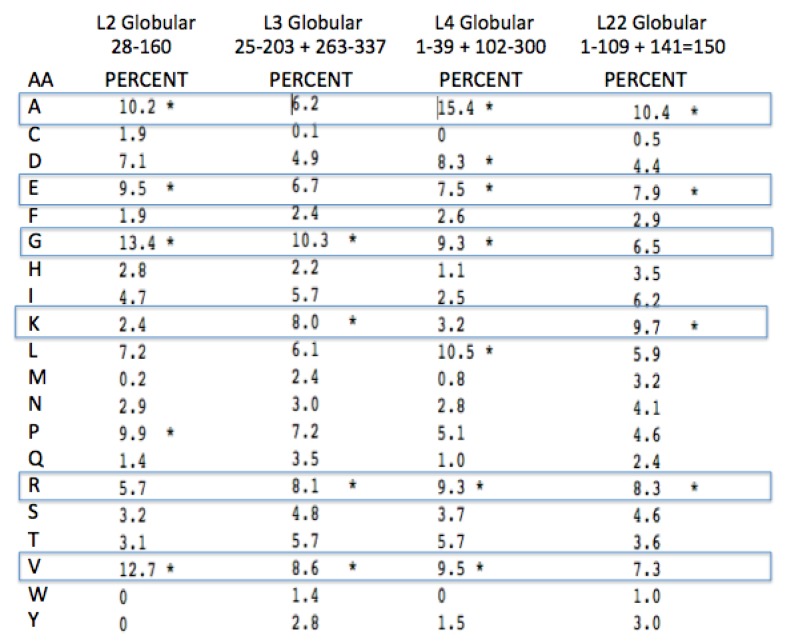
PTC-associated protein globular domain amino acid conservation (see Figure 8 for interpretation).

Given the four L2, L3, L4, and L22 peptide extensions are representative of the earliest peptides, any amino acid compositional bias should hold “fossil” information about the earliest encoded amino acids. In fact, there is a clear amino acid bias seen by Steitz and colleagues [24] in their comparison of the composition of the LSU protein globular domains with their extensions. This analysis, while not restricted to the extensions associated with the PTC, still showed a relative overabundance of Glycine, Lysine and Arginine. We have done an analysis similar to that of Klein *et al.* [24] of the amino acid frequencies conserved in the LSU protein extensions compared to their globular domains. The focus was on the extensions of L2, L3, L4, and L22 found in the Archaea. The clear bias among these is shown in Figure 8 and Figure 9.

Glycine, Arginine and Proline are among the four most common amino acids in all four proteins extensions (Figure 8). Lysine and Alanine are among the top four amino acids in two cases and in the top five amino acids for all four. Among all four of these protein extensions, the sum total for all of the other 15 amino acids common to the alignment pairs is less than 33%. In other words Glycine, Proline, Alanine, Arginine, and Lysine made up over 67% of the distantly conserved amino acids within these PTC associated protein extensions. 

A more complete examination of the amino acid conservation among the LSU protein extensions shows other interesting trends. All of the extensions far from the active site (PTC) have similar amino acid biases, however they include significantly higher amounts of Valine, Serine, the aromatics, Tyrosine and Tryptophan, and even a few negatively charged amino acids. The inclusion of these latter amino acids provides additional information on those proteins relative age. As Valine became available it would have been expected to often substitute for Alanine. The large aromatics would be expected to contribute to rRNA binding through base stacking and intercalation. Extensions containing some negative amino acids, such as Glutamic and Aspartic acid, would allow for positive metal chelation, e.g., Magnesium.

One expects both positive amino acids and amino acids with a high turn propensity, (e.g., Glycine and Proline) in peptides selected to intercalate and stabilize negatively charged RNA. The amino acids in the ribosomal globular protein domains, while still showing some bias for Glycine (largely in turns), show much less of a bias for the other three amino acids (Alanine, Proline, and Arginine) along with much higher values for the hydrophobic and negatively charged amino acids. See Figure 9.

## 8. The GC Code and Arginine and Lysine

This compositional conservation of the PTC-associated protein extensions can now be interpreted to support the hypothesis that the five amino acids; Glycine, Alanine, Proline, Arginine, and Lysine are the potentially primitive amino acids. Interestingly four of these five are encoded today by GC codons. However, it seems highly unlikely that Arginine, let alone Lysine, were available as a very early amino acid, particularly given their metabolic metric distance from the central metabolism. Even though Arginine is coded by a GC triplet codon, it is joined to its tRNA by a Class I Aminoacyl-tRNA synthetase while the other three amino acids use Class II synthetases. There are a couple of possibilities here: One, the first encoded positive amino acid need not to have been Arginine or Lysine, but a simpler structure such as 2,3 diamino-propionic acid or Ornithine, and second, since Lysine is a unique amino acid in being charged by both Classes of Aminoacyl-tRNA synthetases it may hint that the charging of a tRNA of an Ornthine or even a smaller positively charged amino acid was originally made by some form of an early proto Class II synthetase. Note that Ornithine is a component of peptide antibiotics synthesized by a non-ribosomal thioester-dependent system. Although Ornithine is also activated by Lys RS, it is normally excluded from the extant Genetic Code by the Lys RS editing function, which prevents charging of Orn to the Lys tRNA [35]. It is worth noting that, LysRS is perhaps the most promiscuous aaRS, as it catalyzes aminoacylation of tRNA^Lys^ with Arg, Met, Cys, Leu, Thr, Ser, in addition to the cognate Lys [35]. Thus, supporting the idea that there was a co-evolution of the early code and that of selection of positively charged amino acids.

Assuming that the metabolic metric is a good indicator of the sequence of amino acid addition to the early code, Glycine, Alanine, Aspartic acid, and Glutamic acid, each being one catalytic step from the citric acid cycle, would be early entrants. Alanine and Glycine fit the idea of the initial code being only a GC code. However, that raises the question of why Glutamic acid and Aspartic acid are not in the earliest Genetic Code. The very concept of evolution suggests natural selection of peptides, and the likely answer is that the earliest peptides were selected to interact with RNA, a negatively charged polymer due to its phosphate backbone. This interaction would exclude Glutamic and Aspartic acid from the primitive ribosomal peptides as negatively charged. Instead they would be likely precursors to positively charged and neutral amino acids, which could interact with RNA. For example, Proline requires three catalytic steps from Glutamic Acid → Glutamate Semialdehyde by reduction → Proline by a spontaneous cyclization and reduction. 

The early GC code could now be considered to encode Glycine, Alanine, Proline, and Ornithine. This raises a second question: if Ornithine was the first positive amino acid, why was Ornithine replaced by Lysine and Arginine? The answer lies perhaps in a beautiful experiment carried out by Padmanabhan *et al.* [36]. The results obtained were published in a paper entitled “Helix Propensities of Basic Amino Acids increase with the Length of the Side Chain.” In this paper, the ability of a substituted Alanine polymer to form an alpha helix was dependent on the length of the side chain in the basic amino acids: “*The helix propensities for these basic amino acids increase with the length of the side-chain in the rank order 2,3-diamino-L-propionic acid < 2,4-diamino-L-butyric acid < ornithine < lysine*”.

**Scheme 1:**
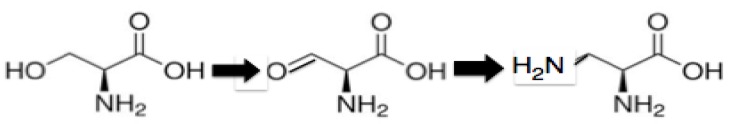
the conversion of serine to 2,3 diamino-L-propionic acid (Diapr).

The PTC contacting extensions found in L2, L3, L4, and L22 are lacking in alpha helices and are dominated by unstructured single-chained peptides, loops with beta turns, and beta hairpins. It would appear then that the early peptides were not only under selection for positive amino acids, but one that would not encourage alpha helical formation. This would have supported an even smaller positive amino acid than Ornithine. There are two candidates: 2,3 diamino-L-propionic acid (Diapr) and 2,4 diamino-L-butyric acid. 2,3 diamino proprionic acid is synthesized from Serine. See Scheme 1.

Serine → 2-amino-3-oxo-proprionic acid by oxidation → 2,3 diamino-proprionic acid by transamination by PLP [37].

While the biosynthesis of 2,4 diamino-L-butyric acid from Aspartic acid (2 steps) is due to a transamination mediated by pyridoxal phosphate of Aspartyl-semialdehyde—similar to the formation as described above of Ornithine from Glutamyl-semialdehyde. The synthesis of 2,4 diamino-butyric acid like Ornithine is closely related to the reduced citric acid cycle as Aspartate is synthesized from oxaloacetate in one catalytic step by a transamination mediated by PLP. The evolution of the positively charged amino acids could now be viewed as the lengthening of the side chain, which correlates with the appearance of the alpha helix in the structure of the evolving peptides: Diapr → 2,4 Diaminobutyric acid → Ornithine → Lysine.

## 9. The Evolution of Protein Structure

The evolution of the Large Ribosomal Subunit is thought to have grown out from the PTC. The metaphor is that of an onion with the PTC at the center, as proposed by Hsiao *et al.* [27], in 2009, who pointed out that “*The conformations of ribosomal protein components near the PT-origin suggest that they are molecular fossils of peptide ancestors whose short length proscribed secondary structure, which is indeed absent from the region of the LSU nearest the PT region*.” The evolution of protein structure has been suggested to have begun with short peptides [38,39] followed by the more complex folds. This evolutionary history of ribosomal protein folds is found in the peptides associated with the PTC followed by the peptides of the ribosomal proteins along the peptide exit tunnel to the ribosomal surface. This evolutionary history is exemplified by the universal ribosomal protein block structure and their extensions, particularly those found at the PTC of the LSU: L2, L3, L4 followed by L22, L23, and L29 of the tunnel.

The PTC contacting extensions found in L2, L3, L4 are of two kinds: single peptides with no secondary structure and peptides that form loops with beta turns. There are no alpha helices in the peptide extensions of L2, L3, L4, and L22. The peptide exit tunnel is dominated by an extension of the protein L22, which is a beta hairpin. The LSU proteins L23 and L29 are on the surface of the LSU at the exit of the peptide tunnel. These proteins show an increase in complexity in their structure, for example in L23 aside from a beta sheet there is the appearance of alpha helices that indicates an increase in the complexity of polypeptide folding, also seen in L29 as a pure alpha helical protein. L23 interacts with the LSU rRNA through a small loop and its globular domain. These latter interactions include positive amino acids at the ends of alpha helices, and more interesting, the beta sheet RNA through Hydrogen bonding via Asn and Gln side chains. 

One thus sees a progression from unstructured positively charged peptides with beta turn loops, and beta hairpins to beta sheets and finally to alpha helices. The addition of alpha helices completes the set of primary protein secondary structural elements. It has been pointed out that beta hairpins are likely precursors to the formation of outer membrane beta barrels and the related OB and SH3 folds [40]. Both such precursors and structures are expected early on, providing membrane permeability and RNA binding. The OB and SH3 folds are known for their binding of nucleic acids [41].

The incorporation of amino acids such as Asn and Gln requires the expansion of the GC code by the addition of AMP (Adenosine monophosphate). The formation of the compact globular domains mediated by alpha helices also suggests the next addition of UMP (Uridine monophosphate) to the code providing the hydrophobic amino acids required to pack the beta sheets and helical secondary structures together. We conclude that the probable evolution of the code from a GC, through a GCA to the full GCAU parallels that for the selection of more complex peptides and proteins.

## 10. Conclusions

**Figure 10:**
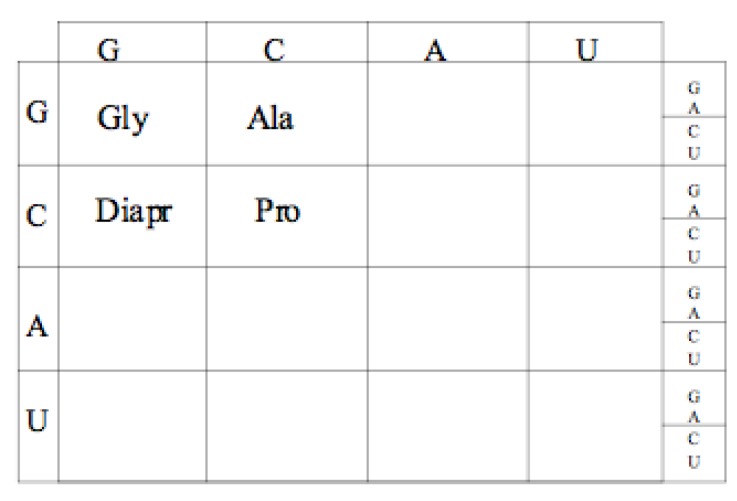
Proposed Guanine Cytosine (GC) code table amino acid assignments.

Our conclusion is that the earliest Genetic Code was a GC code, coding for Glycine, Alanine, Proline and Diamino proprionic acid (Diapr) as shown in Figure 10.

There are a number of early peptides and structural types that would be produced by such and early GC code. The short largely unstructured positively charge peptides, similar to those seen in the extant PTC immediate neighborhood, are clearly expected. Glycine, Proline, Dipar, and Alanine would produce no alpha helices, limited beta hairpins, yet could intercalate into the negatively charged RNA providing some neutralizing stability. Short stretches of alternating Diapr and Alanine and with small clusters of Glycine and/or Proline (beta Turns) would form beta hairpins. These could be ligated to form larger structures including small beta barrels. A run of five or six Glycines with a Diapr at the carboxyl end has the potential to form bi-layered membranes [42,43]. This idea of short peptides interacting with RNA and forming membranes is consistent with an earlier proposal that the earliest form of the peptidyl transfer center (PTC) of the proto-ribosome was composed of a few RNA helices stabilized on a peptide membrane [44].

### 10.1. GCA Code Expansion

**Figure 11:**
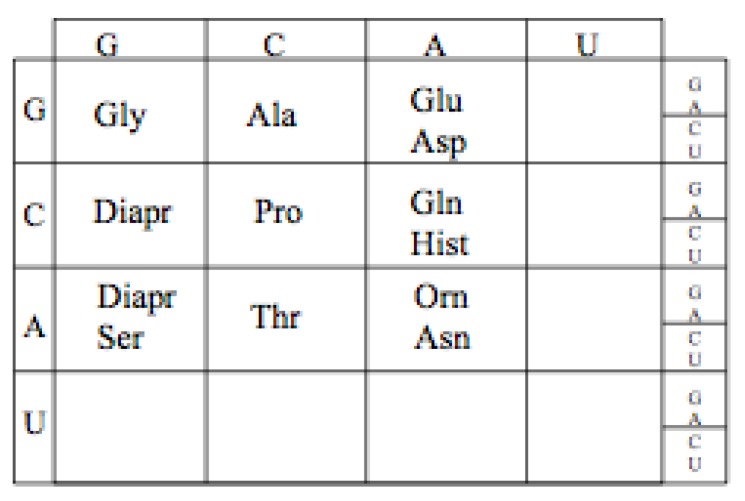
The proposed expanded Adenosine code assignments.

The likely order in which the amino acids, Aspartic acid and Glutamic acid entered the Genetic Code is determined by their aminoacyl tRNA synthetases. Assuming, as pointed out earlier, that the Aminoacyl tRNA synthetases II preceded Aminoacyl tRNA synthetases I, Aspartic acid would have enter the GCA code before Glutamic, Figure 11. This is not independent of the discussion above of the early evolution of the earliest proto-tRNA synthetases.

The Class II aminoacyl tRNA synthetases are distinguished from the Class I aminoacyl tRNA synthetases by their catalytic sites where the amino acid is joined to the tRNA. This is where the operational code is read. The anticodon is read not at the catalytic site of the aminoacyl tRNA synthetase, but at a second site, the anticodon recognition site. The anticodon recognition site of the aspartyl-tRNA synthetase is for example, an OB domain composed of five beta strands as a series of short beta hairpins. The OB domain is also found in a number of translational related proteins including IF2, IF1, EFTu, and EFG. In EFTu and EFG the second domain is an OB domain, which binds the CCA arm of the tRNA. The OB fold is found in the ribosomal proteins S1, S12, S17, and L2 again interacting with RNA. 

The anticodon recognition site of the Glutamyl-tRNA synthetase (in Archaea and Eukarya) is an SH3 fold, which is a related fold to the OB fold domain as it is a similar small five-stranded beta barrel, but with a different topology. It is also found in the translational GTPases, EF-Tu, EF-G, IF2, and the ribosomal proteins L10 and L24. The EFTu’s SH3 module binds the double-stranded RNA in the acceptor arm of the tRNA.

When these two simple protein domains are viewed as assembled from a set of beta hairpins, it supports the idea that protein evolution occurred by ligation from short peptides. In fact, such short beta hairpins are formed by amino acids expected to be encoded by the early form of the genetic code [45]. It is likely that Asparagine was the first to join the genetic code and was followed by Glutamine. There is a clear difference in their compatibility to form alpha helices. The former helical propensity difference is related to the chain length (Asp and Asn *vs.* Glu and Gln), in a manner similar to the relative propensities of Diapr, Ornithine and Lysine, as noted above. 

Serine- (AGC), Threonine- (ACA, ACG ACC) and Histidine -(CAC) would have also entered the code following inclusion of Adenosine monophosphate. The fact that Serine (AGC) shares a codon with Diapr (AGG, AGA) is due to their similarity of structure and that Diapr is synthesized from Serine. Serine and Histidine are at the center of enzymes that are peptidases and may have entered the code as members of early peptidases. If one can only form peptides without being able to hydrolyze them back to their amino acids, the system could ultimately result in a peptide gel.

It is at this point in the evolution that we see evidence of a Class I synthetase (Glutamic Acid and Glutamine) that can now be contrasted to the Class II synthetases. The distinct and variable domain organization of the Class II synthetase, especially those of Glycine, Alanine and Proline, supports the idea of a unique and independent evolution beyond the operational code. There are two exceptions to this independence: One, Histidine appears to have inherited its synthetase structure from that of Glycine, and second, Aspartic acid, Asparagine and Lysine all have very similar Class II synthetases including an OB beta barrel anticodon recognition N-terminal domain. The Class I synthetases have completely different catalytic domain, a Rossmann fold, which is split into an N- and C-terminal domain. Among the Class I synthetases, the Glutamic acid and Glutamine synthetases are in general much more similar to each other than to other Class I synthetases, particularly in terms of the anti codon recognition domain. However, there are two Glutamyl tRNA synthetases that differ only in their anticodon binding domain: one similar to the small alpha beta domains seen in some Class IIs, and the other with a small SH3-like beta barrel.

### 10.2. The Extant Code

**Figure 12:**
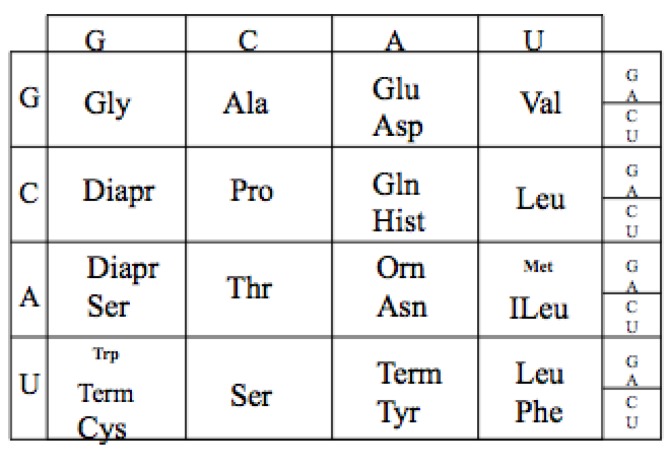
The fully expanded Genetic Code.

The final expansion of the code involved two steps, first the inclusion of Uridine monophosphate in the “reading code” and Adenosine monophosphate in the anticodon thus completing the three nucleotide codon table, Figure 12. The second is the late entry of a final set of amino acids. The presumed continuing selection for more complex proteins, with compact globular domains of hydrophobic cores, required the expansion of the code to include a range of hydrophobic amino acids. These are now encoded by codons with U the middle position of the codon encoding of Valine, Leucine, and Isoleucine. These three are often mischarged, as reflected in their tRNA synthetases containing the Class I editing domains.

Except for Arg (Diapr), the six codons of Leucine and Serine reflect their abundance in extant proteins. This along with the stop codons may reflect a late selective adjustment to the Uracil first position of the codons. It has generally been assumed that Methionine, Phenylalanine, Tyrosine, Tryptophan, and, perhaps, Cysteine were late code additions. In fact the introduction of Methionine and Tryptophan required the complete breaking of the codon wobble degeneracy, as likely one of the last significant coding modification. 

Now an examination of the first position Uracil row can be seen to contain at least three of the assumed late amino acid additions, and the stop codons. Perhaps the entire first position Uracil codons were all stops or unassigned initially? Was the initial translation only statistical in peptide length distribution? Without specific stop and start (Methionine) signals this seems likely. Clearly then, along with start codons, there was selection for stop codons. While multiple stops may have been selected for, there was only one start codon required. Once, started it may be useful or even necessary, as observed in most bacterial genes, to have more than one stop for “insurance”.

Assuming there was selection for increased availability of Serine and Leucine and for the introduction of aromatic amino acids, the obvious extra (or perhaps as yet unused) Uracil first position codons would be available. Thus, it can be assumed that under such conditions this selection exploited the availability of extra stop codons, in a manner similar to that done experimentally today, using amber and opal suppressors [46]. Tyrosine and Tryptophan’s synthetases are very similar and contain a near identical dimerization domain, supporting a similar time of entrance into the code. Cysteine and Methionine have related Class I synthetases similar to those of Glutamic acid and the Class I Lysine synthetase. Phenylalanine’s codon is interesting in that it is in the Uracil middle position column as well, along with the other major hydrophobic amino acids. This may reflect in part selection for some of the code’s hydrophobic substitution error resistance. Phenylalanine’s late entry into the code is also seen in its tRNA synthetase, which appears to have been assembled from components from multiple other preexisting synthetases [47].

## 11. Summary

The Genetic Code began as a GC code recorded in the Operational code stored in the acceptor arm of the tRNA and read by an amino acid charging system. The entry of the amino acids into the expanding Genetic Code following this early code are closely related to their catalytic distance from the Citric Acid (Metabolic Metric) and the increasing protein structural needs. It is in the proteins of the ribosome that the “fossil” record of the evolution of the Genetic Code is most apparent. Clearly the expansion of the Code involved the expansion of the amino acid carriers, tRNAs, also that of the mRNA system and finally that of the modern tRNA synthetases. The latter in particular required multiple domain protein complexes to be able to read both the assumed older information in the acceptor stem and in the anti-codon loop. Such complex proteins required the expanded code coupling the evolution of protein structure to that of the code.

The memory of the GC code is also found in the peptide extensions of the Universal ribosomal proteins, L2, L3, and L4 of the large ribosomal subunit. These ribosomal proteins have peptide extensions that are found at the RNA center of the large ribosomal subunit (LSU)-*the Peptide Transfer Center (PTC)*. The evolutionary expansion of the LSU RNA from the PTC can be correlated with the expansion of the code (GC—GCA → GCAU) as seen in the structure and amino acid composition of the peptide extensions at the RNA of the PTC followed by the beta hairpin extension of L22 interacting with the RNA of the tunnel and finally with the proteins, L23 and L29, and their interaction with RNA at the exit of the tunnel. We conjecture that the evolution of the secondary structure of the polypeptides began with: (1) loops with beta turns; (2) followed by beta hairpins; (3) followed by alpha beta proteins; and then by (4) alpha proteins. The evolution of the secondary structure of the polypeptides correlates quite well with the expansion of the Genetic Code GC → GCA → GCAU and the entry of the amino acids into the evolving code.

From our study of the LSU we have concluded that the proto LSU was composed of an RNA core containing the PTC and was stabilized by peptides made of Gly, Ala, Pro, and Diapr. This collaboration between RNAs and peptides formed an assembly line for the production of peptides from charged micro-helices, which had an operational GC code in their “acceptor” arm. This led to the GC code as the earliest form of the translational genetic code. The evolution of the GC code to one related to the anticodon is based on the appearance of a postulated microhelix that was a stem loop structure that had 17 nucleotides with seven nucleotides in the loop and ten nucleotides in the double helical arm. The XCCA was a remnant from an earlier form of the proto t-RNA [46]. If we add the four nucleotides of XCCA to the 17 nucleotides of the stem loop structure, we get 21 nucleotides. The cloverleaf tRNA likely evolved from these microhelices by ligation [47]. One amusing speculation then is that these proto-RNAs are the precursors of the micro-RNAs, Piwi-RNAs and Si-RNAs and that they occur primarily in the precursor to the eukaryotic cell. The OB fold and the SH3 fold are found in the initiation and elongation proteins, ribosomal proteins and in some of the tRNA synthetases. This would implicate these two folds in the early evolution of the translational apparatus. Furthermore, the evolution of the OB fold and SH3 folds from the beta-hairpins is another case for the evolution of complexity by means of ligation. 

The world from which the ribosome evolved appears to have been one of RNA microhelices and peptides. That is the world we must explore both theoretically and experimentally. “*We shall not cease from exploration, and the end of all our exploring will be to arrive where we started and know the place for the first time*” Mark Twain. 

## 12. Methods

In general the information presented here was from the referenced literature and the sequence (NCBI) and structure (pdb) databases. The analysis of probable ancient amino acids preferences still identifiable in ribosomal proteins and their extensions was done in a somewhat novel manner. Firstly, by combining the results of earlier studies of the Taxonomic Sequence Block structure of ribosomal proteins [21] with the currently available three-dimensional structures showing that the ribosomal protein extensions are contained within such blocks. This meant that these extensions are in general alignable, at least over the majority of their lengths. Rather than generating global alignments used for standard phylogenetic trees among these extensions, we began by generating a very large sets of pairwise alignments. This was done among the Archaea ribosomal proteins, separately for their extensions and their globular domains. In particular, with a focus on the LSU’s active site (PTC) associated proteins and their extensions. Given the great antiquity of these sequence the optimal pairwise alignment were often not consistent with attempts at the more standard global alignments over the very large sets available. Attempts at full global alignments did not include many common residues in either unaligned regions or probably misaligned regions. Secondly, it they did not include as common residues those conserved only along one particular Archaeal line of decent among only pairwise alignments. This is important given the probable last common ancestor for some of these Archaea representatives is on the order of two billion or more years old. Thus, it seems likely that different “fossil” sequence information may have been preserved along these different lines of descent.

In order to ensure the broadest range of comparisons, all pairwise alignments were examined in order to remove those sequence matches between apparently closely related Archaea; those with an over all 75 or higher percent sequence identity. This left between 80 to 120 pairwise alignments for each ribosomal protein and/or associated extension. Many of these remaining pairwise alignments were of partial length and often not over the same partial segment. 

A set of seed sequences were then chosen based on the least similar sequences from the above pairwise initial searches. Thus, these search seed sequences are a set of maximally dissimilar or distinctly related representative. These seeds sets contained five to ten sequences, depending on the overall conservation of that particular protein and/or its extension. At least one of these seed sequences was chosen with known three-dimensional structure. The latter ensured that we had the correct boundaries between the peptide extensions and globular domains. Each seed sequence for each protein globular domain and each extension was then search again with Blast (NCBI) against all available Archaea to produce a second set of pairwise alignments. For each ribosomal protein these were combined, again after highly similar pairwise alignments were again removed. This generated a pairwise alignment data set for each protein’s globular and extension domains. From these data sets, the number of times each of the 20 amino acids was common or shared in an alignment between the seeds and searched sequences was recorded. The number of alignments varied slightly for different LSU proteins from just under one hundred to nearly 300 hundred, as these proteins are not all identified in the same set of Archaea found in GenBank (NCBI). These data were then converted to a percentage normalized to the total number of all pairwise common amino acids for each protein domain or extension. 

In one case, that of L22 extension (118–140), the sequences the traditional over all were conserved enough that a global multi-alignment gave near identical results to the above approach. As a further test, this approach was use on a set of known related proteins of known phylogenetic history, the vertebrate haemoglobins. In that case, there was over a 0.95 correlation between the percentages obtained by the above method and the percentages of each amino acid conserved at 90 percent in a full global alignment. When compared with an attempt at a global alignment of all of the LSU L4 protein extensions, the correlation coefficient dropped below 0.70 and the global alignment appeared to fall apart near the N-terminal, thus supporting the fact that more information appears be obtained from very ancient proteins arising from very deeply related but distinct lines of decent, by this simpler pairwise common statistic. 
